# Sarcopenia and Comorbidity in Gastric Cancer Surgery as a Useful Combined Factor to Predict Eventual Death from Other Causes

**DOI:** 10.1245/s10434-018-6354-4

**Published:** 2018-02-05

**Authors:** Kazuya Kuwada, Shinji Kuroda, Satoru Kikuchi, Ryuichi Yoshida, Masahiko Nishizaki, Shunsuke Kagawa, Toshiyoshi Fujiwara

**Affiliations:** 1 Department of Gastroenterological Surgery, Okayama University Graduate School of Medicine, Dentistry and Pharmaceutical Sciences, Okayama, Japan; 2 Center for Innovative Clinical Medicine, Okayama University Hospital, Okayama, Japan; 3 Minimally Invasive Therapy Center, Okayama University Hospital, Okayama, Japan

## Abstract

**Background:**

Sarcopenia is recognized as an important prognostic factor in various types of cancer, including gastric cancer. While long-term survival analyses typically focus on overall and disease-specific survival, death from other causes has received far less attention.

**Methods:**

We reviewed medical records of 491 gastric cancer patients who underwent gastrectomy from January 2005 to March 2014 and whose preoperative computed tomography (CT) images were available for evaluation of sarcopenia. Sarcopenia was defined as the SMA/BSA index (skeletal muscle area divided by body surface area) below the sex-specific lowest quartile.

**Results:**

Sarcopenia was significantly associated with age, high body mass index (BMI), presence of comorbidity, high American Society of Anesthesiologists physical status (ASA-PS), high T score, advanced stage, large blood loss, and long hospital stay, but was not significantly associated with postoperative complications. Univariate and multivariate analyses of prognostic factors for overall survival revealed that sarcopenia is an independent predictor of poor prognosis [hazard ratio (HR) 1.46, 95% confidence interval (CI) 1.01–2.09, *p*  =  0.0454]. Our analysis of death due to other causes found that non-gastric cancer-related deaths were more frequent among sarcopenia patients with comorbidities than in the rest of our study population (*p*  =  0.0001), while univariate and multivariate analyses revealed that sarcopenia with comorbidity was an independent risk factor for non-gastric cancer-related death (HR 1.84, 95% CI 1.31–3.61, *p*  =  0.0308), as was age.

**Conclusion:**

For gastric cancer patients, sarcopenia increases the risk of death from other causes following surgery, which reveals the importance of developing treatment strategies based not only on cancer status but also on other clinical factors, including sarcopenia and comorbidity.

**Electronic supplementary material:**

The online version of this article (10.1245/s10434-018-6354-4) contains supplementary material, which is available to authorized users.

Sarcopenia is defined as the degenerative loss of skeletal muscle mass, quality, and strength. It is associated with aging and leads to decreased body function and decreased quality of life (QOL).^1^ Since 2010, when the European Working Group on Sarcopenia in Older People first advocated the diagnostic criteria of sarcopenia based on muscle mass, muscle strength, and physical performance,^2^ sarcopenia has been acknowledged as an important factor for cancer patients as well as elderly people. Sarcopenia is associated with an elevated risk of undesirable surgical outcomes such as increased postoperative complications and prolonged hospital stay,^3^^–^5 and is also associated with poor prognoses for several types of cancer.^6^^–^8

Gastric cancer is the third leading cause of cancer death and the fifth most common cancer in the world, with half of all cases occurring in Eastern Asia, including Japan.^9^ Advanced gastric cancer interferes with nutrition through both cancer-associated cachexia and direct obstruction to the passage of food.^10^ Even in early gastric cancer, decline in both weight loss and associated QOL often occur following surgery as it may reduce the stomach’s capacity and consequently decrease food intake.^11^ For these reasons, gastric cancer may be more directly correlated with sarcopenia than many other cancers. Several studies have shown that gastric cancer patients with sarcopenia at the time of surgery experienced worse long-term outcomes than non-sarcopenic patients.^12^^–^15

In the setting of an aging population, many cancer patients receiving surgery present with a variety of comorbidities, such as hypertension, diabetes, cardiovascular disease, brain disease, respiratory disease, renal failure, and mental disorders, as well as typical age-associated declines of basic physiological functions.^16^ Therefore, in clinical practice we must develop treatment strategies for cancer patients that are based not only on cancer stage but also on other patient characteristics, such as age, performance status, comorbidities, and sarcopenia.

In this study, we present a retrospective investigation of the influence of sarcopenia on prognoses of gastric cancer patients who underwent gastrectomy. In addition, we assessed the influence on prognosis of the combination of sarcopenia and comorbidities, focusing particularly on non-gastric cancer-related deaths following surgery. While data related to cancer deaths tend to receive adequate attention in the medical scientific community, comparatively little consideration is given to mortality associated with non-cancer causes of death.^17^ We expect our findings to help inform the development of improved treatment strategies for gastric cancer patients.

## Methods

### Patients

We reviewed the records of 491 patients with gastric cancer who underwent gastrectomy between January 2005 and March 2014 in Okayama University Hospital for whom adequate computed tomography (CT) images taken before surgery were available. This study was approved by the Okayama University Hospital Institutional Review Board.

### Definition of Sarcopenia

The total cross-sectional skeletal muscle area (SMA) at the third lumbar vertebra on preoperative CT images (Hounsfield units of − 30 to 150 for the muscle compartment) was measured using the Synapse Vincent volume analyzer (Fujifilm Medical, Tokyo, Japan) (electronic supplementary Fig. 1a). Then, for normalization, the SMA was divided by body surface area (BSA) to yield the SMA/BSA index (cm^2^/m^2^). Sarcopenia was defined as an SMA/BSA value less than the sex-specific lowest quartile of SMA/BSA; the cut-off values for males and females were 69.7 and 54.2 cm^2^/m^2^, respectively (electronic supplementary Fig. 1b).

### Clinical Data

Patient characteristics included age, sex, height, weight, body mass index (BMI), preoperative comorbidity, and the American Society of Anesthesiologists physical status (ASA-PS) classification. The Charlson Comorbidity Index was used for the evaluation of preoperative comorbidity,^18^ and a score of 1 or higher, with the exclusion of gastric cancer as a comorbidity, was defined as the presence of comorbidity. Histological type was classified into differentiated and undifferentiated, and depth of tumor invasion (T score), lymph node metastasis (N score), and stage were described according to the 3rd English edition of the Japanese Classification of Gastric Carcinoma.^19^ Surgical outcomes included operation procedures such as distal gastrectomy (DG), total gastrectomy (TG) and proximal gastrectomy (PG), reconstruction procedures such as Billroth-I (B-I), Roux-Y (RY) and esophagogastrostomy (EG), and operation time, blood loss, postoperative complications classified according to the Clavien–Dindo classification,^20^ and duration of postoperative hospital stay. Long-term survival data were obtained from medical records.

### Statistical Analysis

Statistical analysis was conducted using JMP software (SAS Institute, Cary, NC, USA). Student’s *t* test was used to assess the continuous variables of age and BMI, and the Wilcoxon signed-rank test was used for the other continuous variables of operation time, blood loss, and duration of hospital stay. Pearson’s *χ*^2^test was used for the categorical variables of sex, comorbidity, ASA-PS, histology, T score, N score, stage, operative procedure, and incidence of postoperative complications, while the log-rank test was used for the Kaplan–Meier survival analyses. Univariate and multivariate Cox proportional hazards regression analyses were performed to assess the effects of prognostic factors on gastric cancer.

## Results

### Clinicopathological Features of Gastric Cancer Patients with Sarcopenia

Sarcopenia was significantly associated with old age (*p*  <  0.0001), high BMI (*p*  =  0.0344), presence of comorbidity (*p*  =  0.0150), high ASA-PS (*p*  =  0.0036), high T score (*p*  =  0.0014), advanced stage (*p*  =  0.0245), large blood loss (*p*  =  0.0012), and long hospital stay (*p*  =  0.0016) (Table 1), but was not associated with postoperative complications, for which, based on univariate and multivariate analyses, age, comorbidity, and blood loss were independent risk factors (electronic supplementary Table 1).

**Table 1:** Clinicopathological features of patients with sarcopenia

	Non-sarcopenia (*n* = 368)	Sarcopenia (*n* = 123)	*p* value
Patient characteristics			
Age, years	66.6 ± 10.7	72.1 ± 8.4	< 0.0001
Sex, male/female (%)	261/107 (71/29)	87/36 (71/29)	0.9676
BMI, kg/m^2^	22.7 ± 3.3	22.0 ± 3.7	0.0344
Comorbidity, + (%)	231 (63)	92 (75)	0.0150
ASA-PS ≥ III (%)	23 (6)	18 (15)	0.0036
Tumor factors (%)			
Histology, differentiated/undifferentiated	207/160 (56/44)	76/47 (62/38)	0.2954
T score ≥ 2	140 (38)	67 (55)	0.0014
N score ≥ 1	116 (32)	50 (41)	0.0557
Stage ≥ III	72 (20)	36 (29)	0.0245
Surgical outcomes			
Operation procedure, DG/TG/PG/others (%)	192/94/38/44 (52/26/10/12)	63/36/11/13 (51/29/9/11)	0.8426
Reconstruction, B-I/RY/EG/others (%)	147/138/37/46 (40/38/10/13)	43/50/10/20 (35/41/8/16)	0.5404
Operation time, min (range)	240 (200–301)	240 (198–294)	0.7045
Blood loss, mL (range)	170 (60–410)	300 (128–550)	0.0012
Post-operative complications, CD ≥ I (%)	80 (22)	36 (29)	0.0888
Post-operative complications, CD ≥ III (%)	31 (8)	14 (11)	0.3293
Hospital stay, days (range)	13 (11–16)	14 (12–19)	0.0016

*BMI* body mass index, *ASA*-*PS* American Society of Anesthesiologists physical status, *T score* depth of tumor invasion, *N score* lymph node metastasis, *DG* distal gastrectomy, *TG* total gastrectomy, *PG* proximal gastrectomy, *B*-*I* Billroth-I, *RY* Roux-Y, *EG* esophagogastrostomy, *CD* Clavien–dindo classification

### Influence of Sarcopenia on Overall Survival of Gastric Cancer Patients

**Fig. 1:**
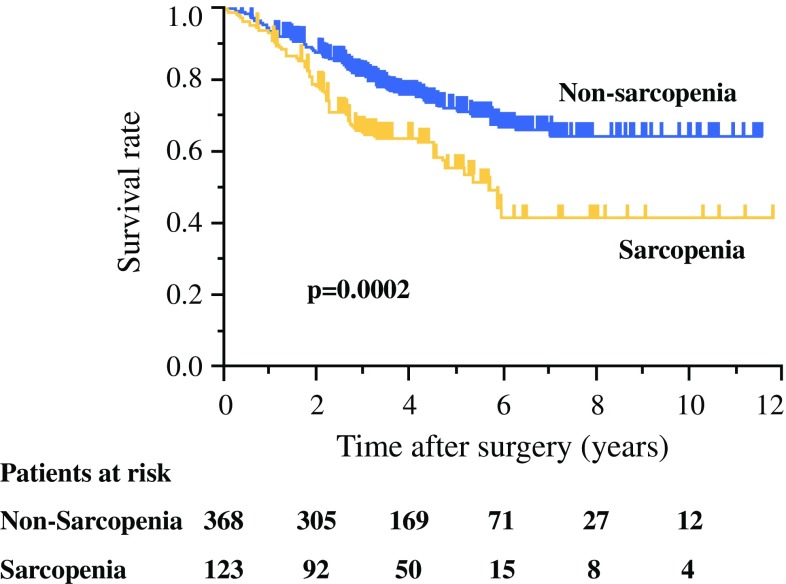
Kaplan–Meier survival curve of gastric cancer patients with sarcopenia

Kaplan–Meier survival analysis showed that 5-year overall survival rates of sarcopenic and non-sarcopenic patients were 56 and 72%, respectively, and that sarcopenic patients had significantly poorer overall survival compared with non-sarcopenic patients (*p*  =  0.0002) (Fig. 1). Univariate and multivariate analyses revealed that sarcopenia was an independent prognostic factor for gastric cancer patients [hazard ratio (HR) 1.46, 95% confidence interval (CI) 1.01–2.09, *p*  =  0.0454], along with age, presence of comorbidity, histological T score, histological N score, and operation time (Table 2).

**Table 2:** Univariate and multivariate analyses of prognostic factors on overall survival in gastric cancer

	Univariate			Multivariate		
	HR	95% CI	*p* value	HR	95% CI	*p* value
Background						
Sarcopenia (+)	1.89	1.34–2.64	0.0004	1.46	1.01–2.09	0.0454
Age ≥ 75 years	1.85	1.32–2.58	0.0005	1.64	1.13–2.37	0.0093
Sex, male	1.00	0.70–1.44	0.9830			
Comorbidity (+)	1.73	1.20–2.55	0.0030	1.70	1.14–2.59	0.0086
ASA-PS ≥ III	1.22	0.62–2.15	0.5406			
Tumor						
Histology (undifferentiated)	1.69	1.22–2.34	0.0016	1.38	0.97–1.96	0.0726
T score ≥ 2	4.27	3.00–6.20	< 0.0001	2.45	1.51–3.99	0.0003
N score ≥ 1	3.65	2.63–5.13	< 0.0001	1.62	1.05–2.54	0.0289
Operation						
Operation procedure (TG)	2.53	1.81–3.51	< 0.0001	1.42	0.96–2.09	0.0789
Operation time ≥ 300 min	1.65	1.14–2.35	0.0080	1.63	1.08–2.43	0.0209
Blood loss ≥ 500 mL	1.92	1.36–2.69	0.0003	0.98	0.66–1.45	0.9316
Post-operative complications (+)	1.61	1.13–2.27	0.0084	1.05	0.72–1.50	0.7937

*HR* hazard ratio, *CI* confidence interval, *ASA*-*PS* American Society of Anesthesiologists physical status, *TG* total gastrectomy

### Influence of Sarcopenia with Comorbidity on Non-Gastric Cancer-Related Death

**Fig. 2:**
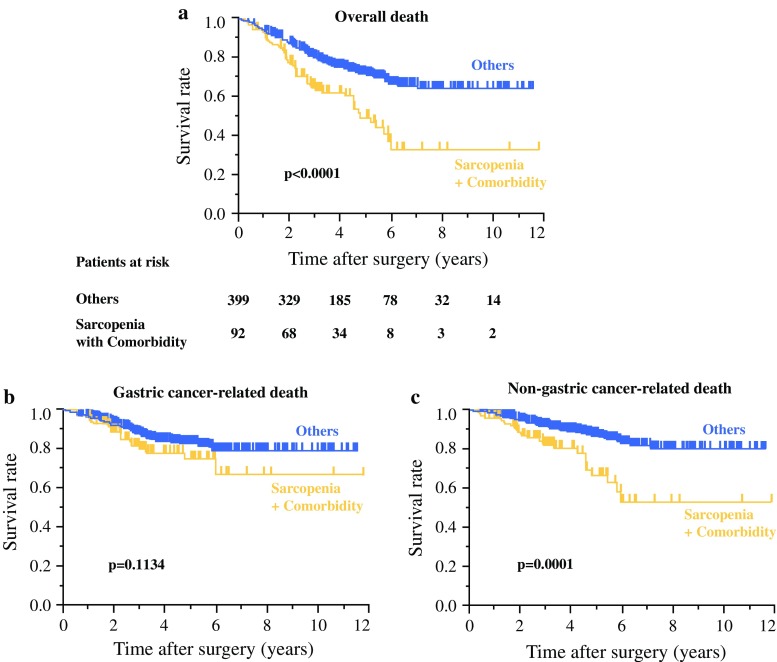
Kaplan–Meier survival curve of gastric cancer patients with sarcopenia and comorbidity based on **a** overall death, **b** gastric cancer-related death, and **c** non-gastric cancer-related death

While sarcopenia and comorbidity were both independent prognostic factors of overall survival in gastric cancer patients (Table 2), neither was an independent risk factor for non-gastric cancer-related death after gastrectomy (electronic supplementary Table 2). When sarcopenia and comorbidity were used as a combined marker, Kaplan–Meier survival analysis showed that sarcopenic patients with preoperative comorbidity had more non-gastric cancer-related death after gastrectomy than the other patients in our study population (*p*  =  0.0001); however, this combined marker was not associated with a significant survival difference for gastric cancer-related deaths (*p*  =  0.1134) (Fig. 2). Of the causes of non-cancer-related death, cardiovascular disease was the most frequent for sarcopenic patients with comorbidity (28%), while pneumonia was the most frequent for other patients (30%) (electronic supplementary Table 3). Multivariate analysis revealed that sarcopenia with comorbidity was an independent risk factor for non-cancer-related death after gastrectomy (HR 1.84, 95% CI 1.31–3.61, *p*  =  0.0308), as was age (Table 3).

**Table 3:** Univariate and multivariate analyses of risk factors for non-gastric cancer-related death

	Univariate			Multivariate		
	HR	95% CI	*p* value	HR	95% CI	*p* value
Background						
Sarcopenia with comorbidity	2.56	1.53–4.19	0.0006	1.84	1.06–3.10	0.0308
Age ≥ 75 years	2.69	1.66–4.32	< 0.0001	2.18	1.31–3.61	0.0028
Sex, male	1.85	1.03–3.62	0.0391	1.61	0.89–3.17	0.1171
ASA-PS ≥ III	1.77	0.74–3.63	0.1845			
Tumor						
Histology (undifferentiated)	0.81	0.49–1.32	0.4100			
T score ≥ 2	1.63	1.01–2.62	0.0439	1.46	0.90–2.37	0.1245
N score ≥ 1	1.14	0.67–1.87	0.6192			
Operation						
Operation procedure (TG)	1.36	0.78–2.27	0.2658			
Operation time ≥ 300 min	1.32	0.74–2.27	0.3336			
Blood loss ≥ 500 mL	1.63	0.97–2.68	0.0668			
Post-operative complications (+)	1.64	0.98–2.68	0.0603			

*HR* hazard ratio, *CI* confidence interval, *ASA*-*PS* American Society of Anesthesiologists physical status, *TG* total gastrectomy

## Discussion

We used CT scans to evaluate sarcopenia as CT results are reliable and accurate. Although CT imaging is costly and invasive, it is particularly appropriate for retrospective studies such as this. SMA at the third lumbar vertebrae is often used for the assessment of sarcopenia.^21^ Although SMA divided by the square of height is used for sarcopenia index in many reports, there is concern that this index may be inappropriate for the detection of sarcopenic obesity, which is associated with higher risks of surgical complications, physical disability, and decreased survival.^22^ A previous report has shown SMA/BSA to be a more useful index for the definition of sarcopenia than SMA/height squared;^23^ therefore, to more effectively detect sarcopenic obesity, we used BSA for normalization and defined the lowest quartiles of the SMA/BSA index as sarcopenia. In this study population, the cut-offs were 69.7 cm^2^/m^2^ for males and 54.2 cm^2^/m^2^ for females, which may be useful as candidate cut-off values for future prospective studies of sarcopenia.

Many reports have suggested that sarcopenia is associated with poor outcomes in both the short and long term,^12^^–^14,24,25 although others found no association between sarcopenia and postoperative morbidity and mortality.^26^ In their recent retrospective study of patients with esophagogastric junction cancer or upper gastric cancer who underwent gastrectomy, Kudou et al. reported that sarcopenia was strongly associated with poor long-term prognosis but not with short-term outcomes such as postoperative complications.^15^ These findings are similar to ours. We found that sarcopenia is not a risk factor for postoperative complications but is an independent prognostic factor. There have been few prospective interventional studies on sarcopenia, but Yamamoto et al. reported that preoperative exercise and nutritional support (median duration 16 days) for elderly sarcopenic patients with gastric cancer eliminated sarcopenia for 18.2% of these patients (4/22) and led to improvement in surgical outcomes.^27^ More prospective studies are needed to evaluate the benefit of exercise and nutritional support for sarcopenic patients, both before and after surgery.

In this study, we also investigated the association of sarcopenia with mortality from causes other than gastric cancer, and, to our knowledge, were the first to do so. We found that sarcopenic patients with comorbidity prior to surgery had a significantly higher risk of non-gastric cancer-related death after surgery compared with those without. However, neither sarcopenia nor comorbidity, when evaluated alone, significantly increased the risk of non-cancer-related death. This result may suggest the possibility that surgical stress worsened sarcopenia and led to non-cancer-related death resulting from comorbidity, and may be more important for patients with early-stage gastric cancer that can be cured by surgery.

While this study has contributed important information for clinical practice, it does have several limitations. First, this was a retrospective, single-center study and may have therefore suffered from selection bias. Second, although we showed the importance of sarcopenia with comorbidity on non-gastric cancer-related death after surgery, comorbidity actually varied from mild to severe, a spectrum that was not considered in our analysis. While ASA-PS is generally considered a good objective indicator of patient health, it did not emerge as a significant risk factor for non-gastric cancer-related death in this study. A better criterion for objective preoperative comorbidity assessment would therefore be helpful in future sarcopenia studies.

Loss of body weight following surgery has recently been identified as a poor prognostic factor for gastric cancer patients as it increases the risk of non-compliance with adjuvant chemotherapy.^28^ The prevention of postoperative weight loss has therefore become an important element for promoting better prognoses. However, Aoyama et al. reported that lean body mass loss was an independent risk factor for decreased adjuvant chemotherapy compliance, which suggests that the prevention of muscle mass loss may be more important than simple prevention of overall weight loss.^29^ In the setting of an aging population, more sarcopenic patients are expected to require surgery, and having a wider and deeper knowledge of sarcopenia and its effects may become very important to future medical management strategies for gastric cancer patients. Our findings emphasize the importance of considering both clinical and pathological factors, and weighing the benefits of surgical interventions carefully when designing a treatment strategy, as maximizing QOL is especially important for patients with low life expectancy.

## Conclusions

In this study, we confirmed that, as several previous studies had reported, sarcopenia is an independent prognostic factor, and, moreover, that sarcopenia in combination with comorbidity is a risk factor for non-gastric cancer-related death after surgery. This latter seems to be a novel finding. Based on these results, we recommend the development of treatment strategies for gastric cancer that are based on both clinical (including sarcopenia and comorbidity) and pathological factors. We also recognize the likelihood that perioperative interventions, such as exercise and nutritional support to improve or prevent sarcopenia, are important for improving prognosis, although prospective studies are required to prove this.

## Electronic supplementary material

Below is the link to the electronic supplementary material.

Supplementary material 1 (DOCX 471 kb)

Supplementary material 2 (DOCX 37 kb)

## References

[1] Rosenberg IH (1997). Sarcopenia: origins and clinical relevance. J Nutr..

[2] Cruz-Jentoft AJ, Baeyens JP, Bauer JM (2010). Sarcopenia: European consensus on definition and diagnosis: report of the European working group on sarcopenia in older people. Age Ageing..

[3] Makary MA, Segev DL, Pronovost PJ (2010). Frailty as a predictor of surgical outcomes in older patients. J Am Coll Surg..

[4] Peng PD, van Vledder MG, Tsai S (2011). Sarcopenia negatively impacts short-term outcomes in patients undergoing hepatic resection for colorectal liver metastasis. HPB (Oxford)..

[5] Lieffers JR, Bathe OF, Fassbender K, Winget M, Baracos VE (2012). Sarcopenia is associated with postoperative infection and delayed recovery from colorectal cancer resection surgery. Br J Cancer.

[6] Antoun S, Lanoy E, Iacovelli R (2013). Skeletal muscle density predicts prognosis in patients with metastatic renal cell carcinoma treated with targeted therapies. Cancer..

[7] Levolger S, van Vugt JL, de Bruin RW, IJzermans JN (2015). Systematic review of sarcopenia in patients operated on for gastrointestinal and hepatopancreatobiliary malignancies. Br J Surg..

[8] Voron T, Tselikas L, Pietrasz D (2015). Sarcopenia Impacts on Short- and Long-term Results of Hepatectomy for Hepatocellular Carcinoma. Ann Surg..

[9] Torre LA, Bray F, Siegel RL, Ferlay J, Lortet-Tieulent J, Jemal A (2015). Global cancer statistics, 2012. CA Cancer J Clin..

[10] Stojcev Z, Matysiak K, Duszewski M, Banasiewicz T (2013). The role of dietary nutrition in stomach cancer. Contemp Oncol (Pozn)..

[11] Takiguchi S, Takata A, Murakami K (2014). Clinical application of ghrelin administration for gastric cancer patients undergoing gastrectomy. Gastric Cancer..

[12] Tamandl D, Paireder M, Asari R, Baltzer PA, Schoppmann SF, Ba-Ssalamah A (2016). Markers of sarcopenia quantified by computed tomography predict adverse long-term outcome in patients with resected oesophageal or gastro-oesophageal junction cancer. Eur Radiol..

[13] Zhuang CL, Huang DD, Pang WY (2016). Sarcopenia is an independent predictor of severe postoperative complications and long-term survival after radical gastrectomy for gastric cancer: analysis from a large-scale cohort. Medicine (Baltimore)..

[14] Huang DD, Chen XX, Chen XY (2016). Sarcopenia predicts 1-year mortality in elderly patients undergoing curative gastrectomy for gastric cancer: a prospective study. J Cancer Res Clin Oncol..

[15] Kudou K, Saeki H, Nakashima Y (2017). Prognostic significance of sarcopenia in patients with esophagogastric junction cancer or upper gastric cancer. Ann Surg Oncol..

[16] Sarfati D, Koczwara B, Jackson C (2016). The impact of comorbidity on cancer and its treatment. CA Cancer J Clin..

[17] Cho H, Mariotto AB, Mann BS, Klabunde CN, Feuer EJ (2013). Assessing non-cancer-related health status of US cancer patients: other-cause survival and comorbidity prevalence. Am J Epidemiol..

[18] Charlson ME, Pompei P, Ales KL, MacKenzie CR (1987). A new method of classifying prognostic comorbidity in longitudinal studies: development and validation. J Chronic Dis..

[19] Japanese Gastric Cancer Association. Japanese classification of gastric carcinoma: 3rd English edition. *Gastric Cancer.* 2011;14:101–12. 10.1007/s10120-011-0041-5 21573743

[20] Dindo D, Demartines N, Clavien PA (2004). Classification of surgical complications: a new proposal with evaluation in a cohort of 6336 patients and results of a survey. Ann Surg..

[21] Prado CM, Lieffers JR, McCargar LJ (2008). Prevalence and clinical implications of sarcopenic obesity in patients with solid tumours of the respiratory and gastrointestinal tracts: a population-based study. Lancet Oncol..

[22] Carneiro IP, Mazurak VC, Prado CM (2016). Clinical Implications of Sarcopenic Obesity in Cancer. Curr Oncol Rep..

[23] Takagi K, Yoshida R, Yagi T (2017). Radiographic sarcopenia predicts postoperative infectious complications in patients undergoing pancreaticoduodenectomy. BMC Surg..

[24] Fukuda Y, Yamamoto K, Hirao M (2016). Sarcopenia is associated with severe postoperative complications in elderly gastric cancer patients undergoing gastrectomy. Gastric Cancer..

[25] Wang SL, Zhuang CL, Huang DD (2016). Sarcopenia adversely impacts postoperative clinical outcomes following gastrectomy in patients with gastric cancer: a prospective study. Ann Surg Oncol..

[26] Tegels JJ, van Vugt JL, Reisinger KW (2015). Sarcopenia is highly prevalent in patients undergoing surgery for gastric cancer but not associated with worse outcomes. J Surg Oncol..

[27] Yamamoto K, Nagatsuma Y, Fukuda Y (2017). Effectiveness of a preoperative exercise and nutritional support program for elderly sarcopenic patients with gastric cancer. Gastric Cancer..

[28] Aoyama T, Yoshikawa T, Shirai J (2013). Body weight loss after surgery is an independent risk factor for continuation of S-1 adjuvant chemotherapy for gastric cancer. Ann Surg Oncol..

[29] Aoyama T, Kawabe T, Fujikawa H (2015). Loss of Lean Body Mass as an Independent Risk Factor for Continuation of S-1 Adjuvant Chemotherapy for Gastric Cancer. Ann Surg Oncol..

