# Supplementary material for: Sarcopenia and Comorbidity in Gastric Cancer Surgery as a Useful Combined Factor to Predict Eventual Death from Other Causes
Source: Ann Surg Oncol. 2018 Feb 5;25(5):1160–6. doi: 10.1245/s10434-018-6354-4 (PMC5891547; doi:10.1245/s10434-018-6354-4)
Supplement: Supplementary file 1 — Supplementary material 1 (DOCX 471 kb) [file 10434_2018_6354_MOESM1_ESM.docx]

Supplementary Figure 1. Definition of sarcopenia

(a) Representative CT scans of patients without and with sarcopenia. Total skeletal muscle area at the third lumbar vertebra was measured using the Synapse Vincent volume analyzer. (b) Histogram of SMA/BSA indices for males and females. Sarcopenia was defined as the lowest quartile of SMA/BSA index for each (yellow area).
