# Supplementary material for: Sarcopenia and Comorbidity in Gastric Cancer Surgery as a Useful Combined Factor to Predict Eventual Death from Other Causes
Source: Ann Surg Oncol. 2018 Feb 5;25(5):1160–6. doi: 10.1245/s10434-018-6354-4 (PMC5891547; doi:10.1245/s10434-018-6354-4)
Supplement: Supplementary file 2 — Supplementary material 2 (DOCX 37 kb) [file 10434_2018_6354_MOESM2_ESM.docx]

Supplementary Table 1. Univariate and multivariate analyses of risk factors for postoperative complications

|  | | Univariate | | | Multivariate | | |
| --- | --- | --- | --- | --- | --- | --- | --- |
|  |  | OR | 95% CI | p value | OR | 95% CI | p value |
| Background | Sarcopenia (+) | 1.49 | 0.93-2.35 | 0.0940 |  |  |  |
|  | Age (≥ 75) | 1.68 | 1.08-2.60 | 0.0219 | 1.62 | 1.01-2.59 | 0.0439 |
|  | Sex (Male) | 1.10 | 0.70-1.77 | 0.6754 |  |  |  |
|  | Comorbidity (+) | 2.11 | 1.32-3.48 | 0.0017 | 1.96 | 1.18-3.33 | 0.0087 |
|  | ASA-PS (≥ III) | 1.56 | 0.76-3.07 | 0.2172 |  |  |  |
| Tumor | Histology (Undifferentiated) | 1.05 | 0.69-1.59 | 0.8303 |  |  |  |
|  | T score (≥ 2) | 1.82 | 1.20-2.78 | 0.0050 | 1.44 | 0.91-2.30 | 0.1222 |
|  | N score (≥ 1) | 1.42 | 0.92-2.18 | 0.1160 |  |  |  |
| Operation | Operation procedure (TG) | 2.05 | 1.31-3.20 | 0.0018 | 1.29 | 0.75-2.17 | 0.3525 |
|  | Operation time (≥ 300 min) | 1.78 | 1.12-2.81 | 0.0155 | 1.54 | 0.91-2.55 | 0.1038 |
|  | Blood loss (≥ 500 mL) | 2.38 | 1.49-3.78 | 0.0003 | 1.86 | 1.11-3.11 | 0.0192 |

Supplementary Table 2. Influence of sarcopenia or comorbidity as a single marker on non-gastric cancer-related death

|  | Univariate | | | Multivariate | | |
| --- | --- | --- | --- | --- | --- | --- |
|  | HR | 95% CI | p value | HR | 95% CI | p value |
| Sarcopenia | 1.89 | 1.14-3.07 | 0.0142 | 1.46 | 0.86-2.41 | 0.1592 |
| Comorbidity | 2.05 | 1.19-3.76 | 0.0090 | 1.45 | 0.81-2.74 | 0.2164 |

Age, sex, and T score were analyzed with sarcopenia or comorbidity for this multivariate analysis based on the results of the univariate analysis in Table 3.

Supplementary Table 3. Cause of non-cancer-related death

|  | Sarcopenia with comorbidity  (n=18) | Others  (n=37) |
| --- | --- | --- |
| Other type of malignant tumor | 4 (22%) | 6 (16%) |
| Cardiovascular disease | 5 (28%) | 5 (14%) |
| Brain disease | 0 (0%) | 4 (11%) |
| Pneumonia | 2 (11%) | 11 (30%) |
| Others | 3 (17%) | 4 (11%) |
| Unknown | 4 (22%) | 7 (19%) |
